# Silica Nanoparticles in Transmucosal Drug Delivery

**DOI:** 10.3390/pharmaceutics12080751

**Published:** 2020-08-10

**Authors:** Twana Mohammed M. Ways, Keng Wooi Ng, Wing Man Lau, Vitaliy V. Khutoryanskiy

**Affiliations:** 1 Reading School of Pharmacy, University of Reading, Whiteknights, Reading RG6 6AD, UK; twana.mohammed@univsul.edu.iq; 2 College of Pharmacy, University of Sulaimani, Sulaimani 46001, Iraq; 3 School of Pharmacy, Faculty of Medical Sciences, Newcastle University, Newcastle upon Tyne NE1 7RU, UK; Keng.Ng@newcastle.ac.uk (K.W.N.); Wing.Lau@newcastle.ac.uk (W.M.L.)

**Keywords:** silica nanoparticles, organosilica, transmucosal drug delivery, mucoadhesion, mucosal penetration

## Abstract

Transmucosal drug delivery includes the administration of drugs via various mucous membranes, such as gastrointestinal, nasal, ocular, and vaginal mucosa. The use of nanoparticles in transmucosal drug delivery has several advantages, including the protection of drugs against the harsh environment of the mucosal lumens and surfaces, increased drug residence time, and enhanced drug absorption. Due to their relatively simple synthetic methods for preparation, safety profile, and possibilities of surface functionalisation, silica nanoparticles are highly promising for transmucosal drug delivery. This review provides a description of silica nanoparticles and outlines the preparation methods for various core and surface-functionalised silica nanoparticles. The relationship between the functionalities of silica nanoparticles and their interactions with various mucous membranes are critically analysed. Applications of silica nanoparticles in transmucosal drug delivery are also discussed.

## 1. Introduction

Among inorganic nanomaterials, silica nanoparticles have attracted a lot of attention in nanomedicine. Silicon-based materials and their oxides (e.g., silica, silicon dioxide, SiO_2_) are appealing as nanomaterials for medical applications because not only do they exist abundantly in nature, but they are also biocompatible. Indeed, silica is “generally regarded as safe” and is approved by the United States Food and Drug Administration (FDA) as an adjuvant (e.g., as an anticaking agent, defoaming agent and emulsifier) in the food industry [1,2,3].

**Figure 1:**
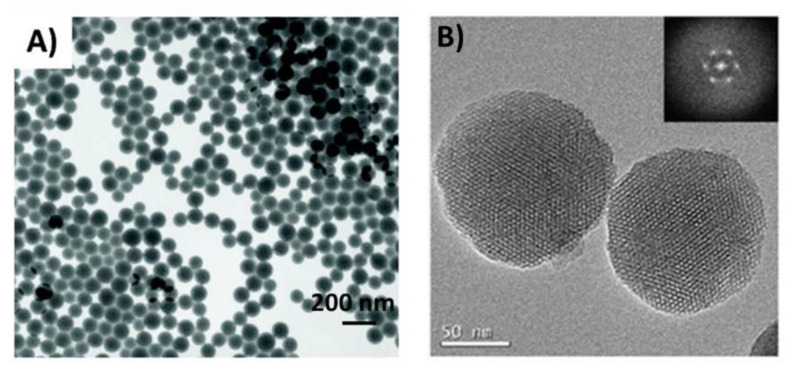
Transmission electron microscopy images of (**A**) nonporous silica nanoparticles (synthesised from SiO_2_ using the Stöber method). Reprinted with permission from [8]. Copyright (2011) American Chemical Society. (**B**) isothiocyanate-functionalized mesoporous silica nanoparticle made of TEOS and 3-aminopropyl triethoxysilane. Reprinted from [9].

Broadly, silica nanoparticles can be classified as nonporous (solid) or mesoporous (pore size: 2–50 nm; Figure 1), both with a similar composition and an amorphous silica structure [4]. The key distinctions are that mesoporous silica nanoparticles have a porous structure, a lower density, and a larger effective surface area [5]. Mesoporous silica nanoparticles are considered as promising nanocarriers for various therapeutic agents, including small molecules, macromolecules, and vaccines which can be loaded into the nanoparticles via physical and chemical adsorption [4,6]. Nonporous silica nanoparticles are also used to load different drugs using encapsulation and conjugation techniques [4]. Depending on the silica source, silica nanoparticles can also be categorised into inorganic nanoparticles or organosilica nanoparticles. Inorganic silica nanoparticles are prepared from pure alkoxysilanes, typically tetraethylorthosilane (TEOS), whilst organosilica are partly prepared from substituted alkoxysilanes [R-Si(OR′)_3_] [7]. These silica nanoparticles can be prepared using relatively simple methods with basic laboratory equipment, and can be easily functionalised using a variety of molecules, such as polymers and fluorescent dyes, to enhance their properties for drug delivery and tracking.

Due to the insolubility of silica and its stability in the harsh gastrointestinal environment containing gastric acid and various proteases, solid silica nanoparticles can potentially be used to protect molecules (e.g., proteins, DNA and RNA) that are liable to degradation in such an environment, and even control their release [6,10]. Solid silica nanoparticles can be advantageous over biodegradable nanocarriers (e.g., polymeric nanoparticles and liposomes) because, in the latter, the drug payload would leach out when they come into contact with the physiological environment, resulting in premature drug release. In most cases, this will lead to a failure of site-specific drug delivery and thus ineffective therapy [10].

Several excellent reviews have previously been published on silica nanoparticles, their synthesis, properties, functionalisation, and various applications [11,12,13]. Mesoporous and nonporous silica nanoparticles have also been extensively reviewed for some biomedical applications [4,14,15,16,17,18]. However, to the best of our knowledge, there is currently no review discussing the application of functionalised silica nanoparticles specifically in transmucosal drug delivery. Considering some interesting studies, trends, and applications that have emerged in recent years, there is a strong need to discuss such an application in a review.

This review provides an introduction to silica nanoparticles, a brief explanation of the common methods of their synthesis, and highlights their potential in transmucosal drug delivery. Additionally, some safety concerns and the in vivo biodistribution of silica nanoparticles are discussed.

## 2. Common Methods of Preparation of Silica Nanoparticles

**Figure 2:**
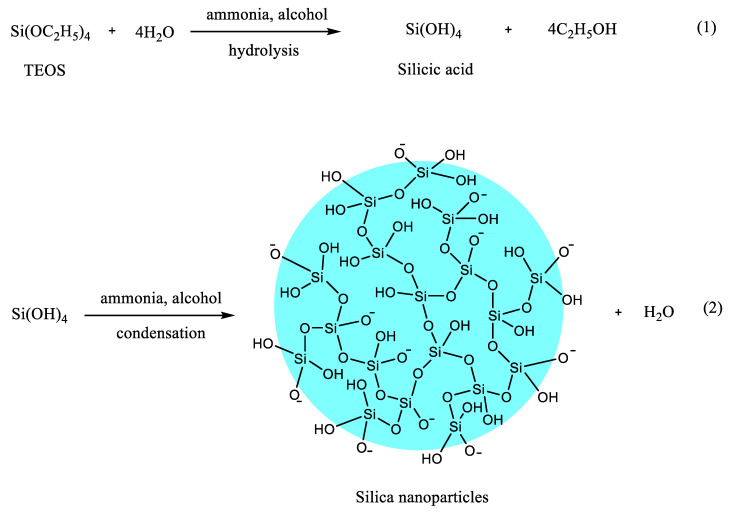
A reaction scheme showing the synthesis of silica nanoparticles from TEOS; Reaction (**1**) includes the hydrolysis of TEOS to form silicic acid, followed by reaction (**2**) which involves the condensation of the silicic acid to produce silica nanoparticles with siloxane bridges (Si-O-Si).

Traditionally, silica nanoparticles are prepared using the Stöber method [19], in which TEOS is used as the silica source, water and ethanol as solvents, and ammonia as a catalyst. This method generally involves the hydrolysis of TEOS to form silicic acid, followed by condensation of the silicic acid to produce silica particles with siloxane bridges (Si-O-Si) (Figure 2). Nucleation and particle growth are involved in the formation of the silica nanoparticles [20,21,22,23,24]. Nucleation is the formation of solid particles (nuclei) from soluble TEOS monomers in a homogenous liquid. Particle growth is achieved either through the addition of TEOS monomers to the nuclei [23,24], or the aggregation of the nuclei to form larger particles [20,25].

Other silica sources, such as tetramethoxysilane (TMOS), tetrakis-2-hydroxyethylorthosilicate, and trimethoxyvinylsilane, have also been used in the synthesis of silica nanoparticles [26]. Several modifications of the Stöber method have been proposed in order to obtain particles with specific physicochemical properties, e.g., size, polydispersity index, shape and surface functionalities [27,28]. The size and shape of the silica nanoparticles can be controlled by tuning the concentration of the precursor, the type of solvent and catalyst used, as well as the reaction temperature [5,27,28,29]. To prepare mesoporous silica nanoparticles, surfactants, including cetyltrimethylammonium bromide (CTAB) and cetyltrimethylammonium chloride (CTAC), are added as structure directing agents to promote the condensation of the silica precursor around the templates. The surfactants are then removed, leaving a porous silica nanostructure [30,31]. Also, pore-expanding agents (e.g., alkanes) are used to increase the pore size which could allow loading of large molecules and the potential enhancement in the loading efficiency of the particles [32]. The size and morphology of the mesoporous silica nanoparticles can be tailored by changing the molar ratios of the silica precursors, the surfactants, or the type of catalysts to produce spherical, rod-shaped, or worm-like particles [5].

**Figure 3:**
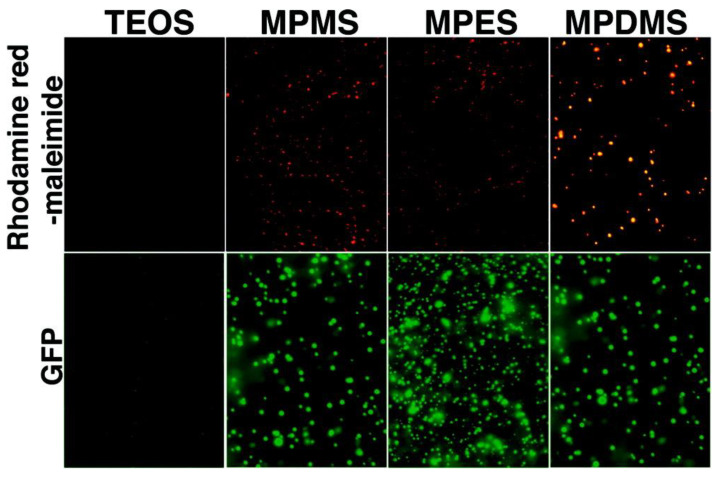
Fluorescence microscopy images of surface modified thiolated organosilica and TEOS nanoparticles. The nanoparticles were mixed with rhodamine red maleimide (upper panels) and GFP (lower panels). TEOS: Tetraethylorthosilane, MPMS: 3-mercaptopropyltrimethoxysilane, MPES: 3-mercaptopropyltriethoxysilane and MPDMS: 3-mercaptopropylmethyldimethoxysilane. Reprinted with permission from [34]. Copyright (2008) American Chemical Society.

Nakamura and Ishimura [33] synthesised thiolated organosilica nanoparticles from an organosilicate (3-mercaptopropyltrimethoxysilane, MPMS) using the Stöber method. Later, they reported the possibilities of forming silica nanoparticles using other organosilicates (3-mercaptopropyltriethoxysilane and 3-mercaptopropylmethyldimethoxysilane (MPDMS)) using the Stöber method (with ethanol) and a complete aqueous synthetic technique (without ethanol) [34]. These organosilica nanoparticles have abundant internal and surface thiol groups, enabling their modification with fluorescent dyes such as rhodamine red, green fluorescent protein (GFP) (Figure 3) and various biomolecules via thiol-maleimide chemistry [35]. However, the authors observed that the organosilica nanoparticles had a wide size distribution [33,34]. In 2010, they found that performing the reaction at a high temperature (100 °C) instead of room temperature narrowed the size distribution of MPMS silica nanoparticles [36]. The hybrid organosilica nanoparticles were synthesised from a combination of MPMS and MPDMS in the presence of sodium dodecyl sulfate as a surfactant [37]. Solid-state ^13^C nuclear magnetic resonance and Raman spectroscopy revealed the presence of disulfide bonds in the structure of these hybrid (MPMS–MPDMS) nanoparticles. Using transmission electron microscopy (TEM), it was found that MPMS–MPDMS organosilica nanoparticles degraded in the presence of glutathione. This degradation manifested as irregular shapes in the decomposed nanoparticles, compared to the regular spheres observed in the absence of glutathione. This could be due to the ability of glutathione to attack the disulfide bonds of the nanoparticles via thiol/disulfide exchange reaction which leads to the oxidation of glutathione itself and the consumption of a portion of the reduced form of glutathione by the nanoparticles as indicated by Ellman’s assay [37]. The glutathione-responsive degradability of MPMS-MPDMS organosilica nanoparticles makes these nanoparticles a promising carrier for targeted delivery of anticancer drugs due to the presence of a higher concentration of glutathione in cancer cells compared to normal cells [37].

It is also possible to synthesise bifunctional silica nanoparticles by combining two organosilicate precursors with different functional groups. For instance, using a nanoprecipitation method, Chiu et al. [38] synthesised silica nanoparticles (~150 nm) with both thiol and amine functionalities from MPMS and 3-aminopropyltrimethoxysilane (APMS). Typically, the organic phase contained MPMS and APMS, DMSO, diethylenetriaminepentaacetic acid (a reducing agent to minimise thiol oxidation) and HCl. This phase was incubated for 24 h to allow acid-catalysed hydrolysis and condensation of organosilanes, forming oligomeric or polymeric silica structures. Then, a small portion of the organic phase was injected into water (aqueous phase) under constant stirring at room temperature which resulted in the formation of silica nanoparticles. It was found that the presence of a small proportion of APMS was necessary to produce colloidally stable nanoparticles. The molar ratio of MPMS/APMS in the reaction mixture was directly proportional to the zeta potential but inversely proportional to the thiol content. As these nanoparticles had positively charged surfaces (their zeta potential ranged from 30 to 50 mV), they bound to antisense oligodeoxyribonucleotide (G3139) efficiently as a result of electrostatic attraction between the cationic nanoparticles and the anionic therapeutic [38].

Silica nanoparticles can also be synthesised using a reverse microemulsion method where the silica source (TEOS or TMOS) is added to a preformed water-in-oil emulsion [39]. Again, here, the size of the nanoparticles can be tuned by the composition and the pH of the aqueous phase, changing the type of emulsifier, water-to-emulsifier ratio, amount of TEOS, and the type of organic solvent comprising the organic phase of the emulsion [40,41].

Irmukhametova et al. [42] have pioneered the formation of thiolated silica nanoparticles (with the size of ~50 nm) from MPMS using DMSO as a solvent and NaOH as a basic catalyst. The mechanism driving the formation of these nanoparticles is believed to be the hydrolysis and subsequent condensation of the methoxysilane groups in MPMS, as well as the formation of disulfide bonds via partial oxidation of the thiol groups in MPMS. Observation of the turbidity of the reaction mixture during nanoparticle synthesis confirmed that air bubbling the reaction mixture is necessary to form stable colloidal thiolated silica nanoparticles. It was hypothesised that the oxygen present in the air could oxidise some of the thiol groups of MPMS, resulting in the formation of disulfide bridges. In the absence of oxygen (but in the presence of N_2_), no significant changes in the turbidity of the reaction mixture were observed. Control experiments performed using a water–ethanol mixture instead of DMSO resulted in milky suspensions of large particles. This could be due to the fact that protic solvents, such as water and ethanol, could facilitate the hydrolysis and subsequent condensation of methoxysilane groups to ultimately result in faster nucleation and particle growth compared to DMSO [42].

Later, Al Mahrooqi et al. [43] investigated the effects of various parameters, including catalyst type and concentration, MPMS concentration and solvent type on the physicochemical properties of these thiolated silica nanoparticles. From the dynamic light scattering (DLS) analysis, it was found that the size of the nanoparticles decreased from ~290 nm to ~50 nm with increasing NaOH concentration (0.05 M up to 0.5 M), but a further increase in the concentration of NaOH (0.5 to 0.9 M) did not significantly change the size of the nanoparticles. Additionally, it was found that the thiol content of the nanoparticles (measured by Ellman’s assay) increased with increasing NaOH concentration. The use of an acidic catalyst (HCl) not only resulted in larger particles, but the particle size increased with increasing HCl concentration (the mean particle size was 1.18, 5.30, and 10.46 µm when 0.1, 0.3 and 0.5 M HCl was used, respectively). With an increasing concentration of MPMS (but only over a limited range of 0.13–0.40 M), a linear increase in the size of the nanoparticles was observed. However, a lower concentration of MPMS (0.04 M) resulted in an increase in the size of the nanoparticles. Further experiments using different organic solvents revealed that increasing the dielectric constant of the solvents decreased the nanoparticles size wherein nanoparticles synthesised using DMSO (with a dielectric constant of 47) had the smallest size (45 ± 3 nm) and low polydispersity index (0.181) [43]. Clearly, the ability to control the physicochemical properties of these thiolated silica nanoparticles can have a significant impact on the behaviour (e.g., mucoadhesion and biodistribution) of these nanoparticles in biological environments.

## 3. Applications of Silica Nanoparticles in Drug Delivery: Loading Capacity and Release

Silica nanoparticles are now extensively used as nanocarriers for the delivery of various drugs having different physiochemical, pharmacokinetic and pharmacodynamic properties. Their main applications in drug delivery include improving the dissolution rate of poorly water-soluble drugs, controlled release, and targeted drug delivery [44,45,46]. Initially, MCM-41 have been used to control the release profile of ibuprofen [47]. The loading capacity and ibuprofen release could be controlled using various functional groups of silica pore wall [47,48]. Generally, the same methods which are used to load drugs into the other nanoparticles are also used for the silica nanoparticles. These include drug loading during the synthesis of the nanoparticles and also after the nanoparticles are formed. However, polymer-based drug delivery systems usually need the organic solvents for drug loading, which cause some toxicological issues [49]. The mechanism of the drug release from silica nanoparticles mainly depends on the diffusion of the drug molecules from the pores of the nanoparticles. However, in polymer-based drug delivery systems the release depends either on the hydrolysis-induced erosion of the polymer or on the swelling of polymeric matrix which is usually occurred upon dispersion of the systems in the biological environments [49]. This will lead to premature drug release and the lack of control over the drug release in the target sites which causes ineffective drug therapy and unwanted systemic side effects. Stimuli-responsive silica nanoparticles release the loaded drug upon both endogenous (e.g., pH, enzyme, redox, and glucose) and exogenous (light, temperature, ultrasound, electric and magnetic fields) stimulation [50]. The ordered porous structure of mesoporous silica nanoparticles allows easy loading of various drugs into these nanoparticles and homogenous distribution of the drugs in the nanoparticles. The size, shape and the surface properties of the pores affect the drug loading capacity, the nanoparticles-drug interactions, the drug release properties and finally the therapeutic activities [5]. Thus, drugs with molecular size smaller than nanoparticles pore size can be loaded into the nanoparticles. For loading proteins and larger macromolecules, nanoparticles with larger pore size are required [5]. The surface properties also determine the nature of the nanoparticles-drug interactions and this can be tuned with the surface functionalisation which includes the introduction of various functional groups at the surface of the nanoparticles leading to the desired surface charge, surface chemistry and the hydrophilic-lipophilic character of the nanoparticles [5]. The nature of the nanoparticles-drug interactions has a significant role in determining the loading capacity and the drug release profile. For instance, Wani et al. [51] demonstrated that methotrexate loading capacity of thiol-functionalised mesoporous silica nanoparticles was significantly higher (18% *w/w*) than both mixed thiol-amine (6% *w/w*) and amine (1% *w/w*) functionalised counterparts. Wani et al. [51] also observed a strong pH dependence of methotrexate release from thiol-functionalised mesoporous silica nanoparticles with a rapid release in acidic pH and a very slow release in neutral pH. In contrast, they observed almost similar methotrexate release profile from both amine and mixed thiol-amine functionalised silica nanoparticles which was rapid and pH-independent. Strong electrostatic interactions between methotrexate and negatively charged thiol-functionalised silica nanoparticles not only increased the drug loading capacity but also significantly decreased the rate of drug release. Decreasing the ionization of the silica in acidic pH weakens the interactions and facilitates rapid drug release [51]. Moreover, Datt et al. [52] showed that amine functionalized MCM-41 provided a slow drug release due to the strong interaction between the amino groups of the nanoparticles and the carboxyl groups of aspirin. However, non-functionalised MCM-41 showed a rapid aspirin release [52]. Many other publications have reported the feasibility of plain and functionalised silica nanoparticles in controlled drug delivery [53,54,55].

## 4. Transmucosal Drug Delivery

Transmucosal drug delivery refers to the administration of therapeutic agents via mucosal membranes. The established routes of transmucosal administration include the oral cavity (buccal, gingival and sublingual) [56], esophagus [57], gastrointestinal tract (GIT) [58], nose [59], eyes [60], rectum [61], vagina [62], and urinary bladder [63]. Transmucosal drug delivery has several advantages including the ease of administration, non-invasive nature, and improved patient compliance. However, there are some obstacles in transmucosal drug delivery, including luminal (pH and enzymes), mucus and epithelial barriers. The strategies of mucoadhesion, mucus-penetration and nanoscale technologies have been used to overcome these barriers [64,65,66,67,68]. Mucoadhesion refers to the phenomenon whereby synthetic or natural materials adhere to mucous membranes [69,70]. The application of mucoadhesive materials first appeared in dentistry in 1947 when Scrivener and Schantz mixed tragacanth gum with a dental adhesive to deliver penicillin to the oral mucosa [68]. This later led to the development of Orabase ^®^ [68] which is commonly used for the treatment of oral ulcer. Later the research of Nagai with co-workers in the 1980s reported the potential of mucoadhesion in drug delivery where polymers such as hydroxypropyl cellulose and Carbopol 934 were used in the design of various dosage forms. These include vaginal discs for delivering bleomycin (an anticancer drug) to the mucosal surfaces of the human cervical canal, discs containing insulin and tablets of triamcinolone acetonide (marketed as Aftach, used for the treatment of aphthous stomatitis), both for administration to the oral mucosa, as well as a powder formulation of insulin for nasal administration [71,72]. Since then, various classes of polymers have been used in the design of mucoadhesive drug delivery systems.

Thiomers are a class of mucoadhesive materials which can be synthesised through the immobilisation of thiol groups on various materials. Some examples of thiomers include thiolated-polycarbophil [73], chitosan [74], pectin [75,76], graphene oxide [77], polyvinylpyrrolidone [78], and hyaluronic acid [79]. Their mucoadhesive properties can be due to their ability to form disulfide bonds with cysteine-rich domains of mucus glycoproteins via thiol/disulfide exchange reactions or oxidation of their thiol groups. In situ cross-linking can be another possible mechanism for the mucoadhesion of thiomers [80].Various dosage forms have been prepared using these thiomers with enhanced mucoadhesive properties. These include micro-and nanoparticles, matrix tablets and eye drops [80]. Several other materials with enhanced mucoadhesive properties including polymers bearing catechol, boronate, acrylate, methacrylate, and maleimide functional groups are developed and were recently discussed by Brannigan and Khutoryanskiy [81].

## 5. Factors Influencing Mucosal Drug Delivery

Researchers are currently investigating two main types of mucosal drug delivery systems which are mucoadhesive and mucus-penetrating formulations. The mucoadhesive formulations are able to adhere to the the mucus layer of the mucosal membranes. To date, six theories explain mechanisms of mucoadhesion and these include electronic, adsorption, wetting, diffusion, fracture and mechanical theory [68]. Mucoadhesive formulations enhance the drug retention time at the site of absorption/action, which can lead to the enhanced drug bioavailability. Mucoadhesive formulations are typically prepared using hydrophilic polymers having ionic and/or non-ionic functional groups with the ability of hydrogen bond formation with the mucus components. They generally show strong physical and chemical interactions with the mucin macromolecules, hardly diffuse through mucus layer, and are trapped in the mucus gel [68]. In contrast, mucus-penetrating formulations do not contain materials with mucoadhesive properties and are non-ionic or net-neutral hydrophilic polymers with stealth properties. They usually do not interact with the components of the mucus (mucus-inert) and can reach the underlying epithelial tissues and deliver the loaded drugs in the desired tissue [82]. Consequently, mucus-penetrating formulations have a short mucosal retention time. Alternatively, the mucus-penetrating formulations can be designed using the concepts of zeta-potential changing [83] and mucolysis [84]. The dosage form-mucin interactions are considered as influencing factors in mucosal drug delivery as they determine whether the formulation is mucoadhesive or mucus-penetrating. The other factors which affect the efficiency of mucosal drug delivery are the physicochemical characteristics of the dosage form and the drug (e.g., particle size, shape and zeta potential) and the physiological conditions, e.g., pH, presence of enzymes, the type of mucosa, the mucus thickness and the mucus turnover rate. Short-chain PEG is a typical example of the polymers used in the design of various mucus-penetrating formulations including nanoparticles [85], liposomes, and micelles [86], but some other materials have also been explored which are discussed by Khutoryanskiy [82]. Both mucoadhesive and mucus-penetrating nanoparticles are desirable in transmucosal drug delivery as each one has its own features and advantages. There is also an emerging trend in formulating nanomedicines using combination of mucoadhesive and mucus-penetrating nanoparticles for the efficient transmucosal drug delivery [87,88,89].

## 6. Applications of Silica Nanoparticles in Transmucosal Drug Delivery

**Figure 4:**
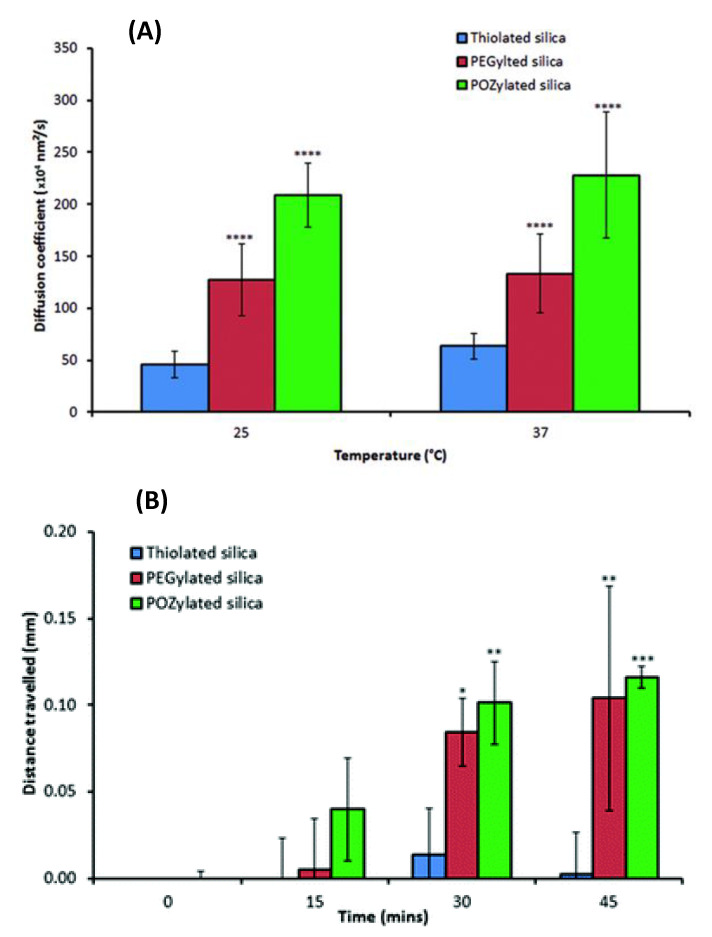
(**A**) Diffusion coefficients of thiolated, PEGylated and POZylated silica nanoparticles in 1% *w/v* porcine gastric mucin suspension determined by nanoparticle tracking analysis at 25 and 37 °C; Data show the mean ± standard deviation, where *n* = 9. (**B**) Penetration of thiolated, PEGylated and POZylated silica nanoparticles into porcine gastric mucosa. The values represent the means of 3 repeats ± standard deviation; all values were subtracted from values obtained for the blanks. *: *p <* 0.05, **: *p <* 0.01 and ***: *p* < 0.005. Reproduced from [96] with permission from the Royal Society of Chemistry.

Silica nanoparticles have a range of potential applications including drug delivery (mucosal and controlled delivery) [51,65,90,91,92], diagnostics [93], and tissue engineering (mainly for bone regeneration) [94,95]. In this section, we will discuss the applications of silica nanoparticles in transmucosal drug delivery. The thiolated silica nanoparticles developed by the Khutoryanskiy group have abundant thiol groups which can be prepared in a single-step process (unlike the nanoparticles based on thiomers which require two steps of synthesising the polymers and then elaborating the nanoparticles from the thiomers thus synthesised). These thiolated silica nanoparticles can be functionalised with polyethylene glycol (PEG) [42], poly-2-ethyl-2-oxazoline (POZ) [96], poly-2-methyl-2-oxazoline, and poly-2-n-propyl-2-oxazoline [97], and hydroxyethylcellulose [98]. Upon functionalisation a significant number of the free thiol groups will be masked by the polymers and therefore would not be available for chemical reactions. Thiolated silica nanoparticles exhibited mucoadhesive properties in vitro on bovine cornea [42], porcine bladder mucosa [99] and rat intestinal mucosa [90]. Their mucoadhesiveness was reduced upon PEGylation (PEGylated silica nanoparticles) [42,99] and POZylation (POZylated silica nanoparticles) [90]. However, these modifications enhanced their diffusion in porcine gastric mucin dispersions and penetration into the porcine gastric mucosa, as measured by nanoparticle tracking analysis and fluorescence microscopy, respectively (Figure 4) [96]. As shown in Figure 4 the diffusion coefficient of PEGylated and POZylated silica nanoparticles is greater than thiolated silica nanoparticles. Also, PEGylated and POZylated silica nanoparticles moved further into the gastric mucosa compared to the thiolated counterpart (Figure 4). Mun et al. [100] showed that neither thiolated nor PEGylated (with 750 and 5000 Da PEG) silica nanoparticles penetrated the intact bovine cornea. They also revealed that thiolated silica nanoparticles did not penetrate the de-epithelialised cornea, which could be due to the interactions of their thiol groups with the cysteine domains of the corneal stroma. Also, PEGylated (with 750 Da PEG) silica nanoparticles did not penetrate the de-epithelialised cornea as they had some remaining thiol groups available for binding with the cysteine domains of the stroma. However, PEGylated (with 5000 Da PEG) silica nanoparticles penetrated the de-epithelialised cornea, which could be due to better coverage of silica particles with a stealth layer of larger molecular weight PEG (compared to PEGylated nanoparticles with 750 Da PEG), decreasing the nanoparticles–cysteine interactions.

Mansfield et al. [97] also studied the effect of thiolated silica functionalisation with three different 5 kDa poly(2-oxazolines): poly-2-methyl-2-oxazoline, poly-2-ethyl-2-oxazoline, and poly-2-n-propyl-2-oxazoline on their diffusion in mucin dispersions and through freshly excised porcine gastric mucosa. They established that alkyl chain variation could substantially affect the ability of these nanoparticles to diffuse through mucosal barriers. Nanoparticles functionalised with poly-2-methyl-2-oxazoline and poly-2-ethyl-2-oxazoline exhibited mucus-penetrating properties, whereas the nanomaterial decorated with more hydrophobic poly-2-n-propyl-2-oxazoline did not show any significant increase in penetration compared to thiolated silica.

**Figure 5:**
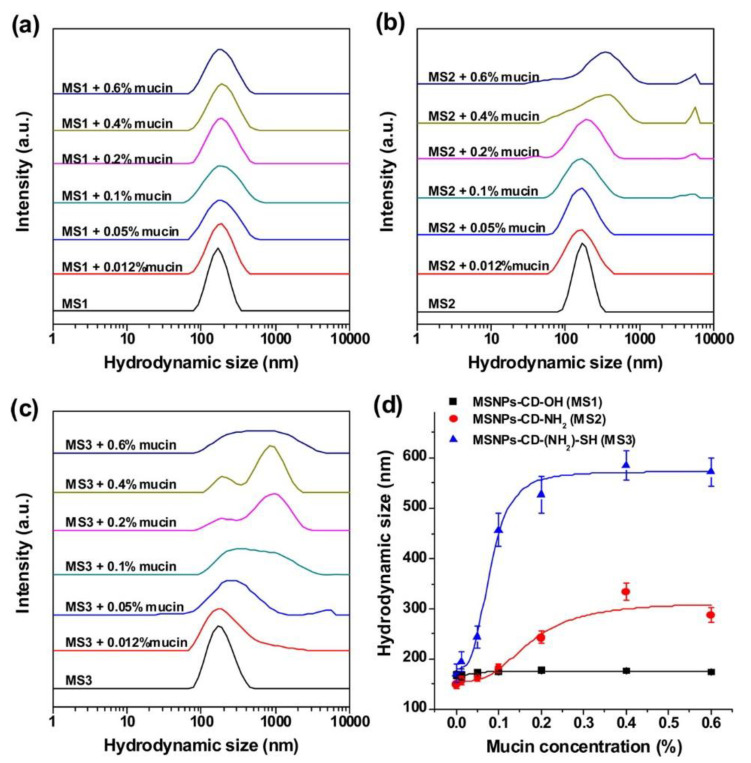
DLS size distribution of (**a**) MSNPs-CD-OH (MS1), (**b**) MSNPs-CD-NH_2_ (MS2) and (**c**) MSNPs-CD-(NH_2_)-SH (MS3) after mixing with different concentrations of mucin dispersed in acetate buffer solution (pH 4.5) for 30 min. (**d**) Effect of mucin concentration on mucin-particle interactions. Reprinted with permission from Zhang et al. [101]. Copyright (2014) American Chemical Society.

**Figure 6:**
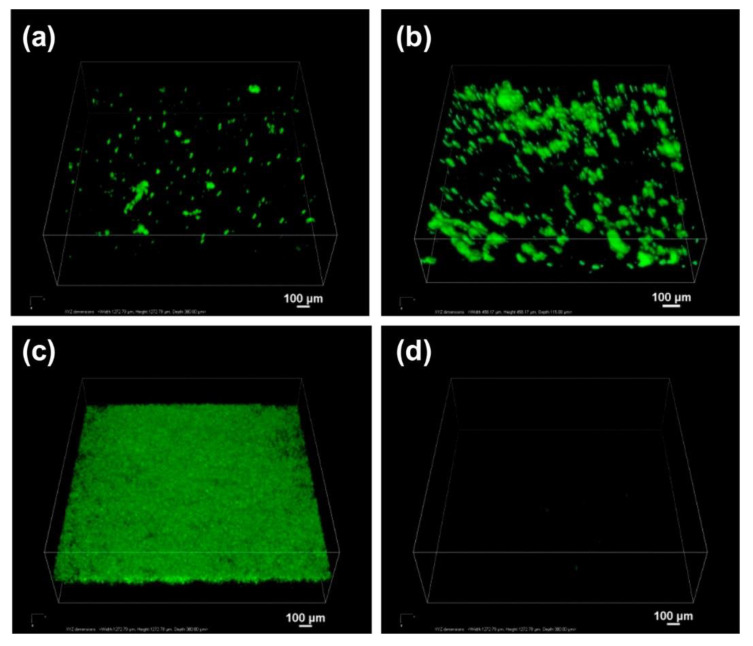
Confocal microscopy volume view images of porcine bladder wall incubated in artificial urine (pH 6.1) containing fluorescein isothiocyanate (FITC)-labelled-(**a**) MSNPs-CD-OH, (**b**) MSNPs-CD-NH_2_, (**c**) MSNPs-CD-(NH_2_)-SH and (**d**) PBS (control) for 2 h. The green fluorescence indicates the presence of FITC-labelled MSNPs on the bladder wall. The data are representative images from three independent experiments. Scale bars are 100 μm in all images. Reprinted with permission from Zhang et al. [101]. Copyright (2014) American Chemical Society.

Zhang et al. [101] synthesised β-cyclodextrin modified mesoporous silica nanoparticles with three different surface functionalities, namely hydroxyl, amino, and thiol groups. These nanoparticles were referred to as MSNPs-CD-OH, MSNPs-CD-NH_2_, and MSNPs-CD-(NH_2_)-SH, respectively. They investigated the mucoadhesive properties of these nanoparticles through particle-mucin interactions by measuring the size of the mixture of mucin suspension and the nanoparticles suspension using DLS (i.e., an increase in the size indicated the presence of mucoadhesive interactions). This was also supported by confocal microscopy of porcine bladder mucosa exposed to fluorescein isothiocyanate-labelled nanoparticles, followed by washing with artificial urine. They found that thiol-functionalised silica nanoparticles had superior mucoadhesiveness compared to both amino- and hydroxyl-functionalised counterparts. This was evident from the greater change in the size of the thiol-functionalised silica nanoparticles compared to the amino- and hydroxyl-functionalised nanoparticles upon mixing with a mucin suspension. The size of hydroxyl-functionalised nanoparticles did not change upon mixing with different concentrations of a mucin suspension. However, a significant increase in the size of amino- and thiol-functionalised nanoparticles was observed (Figure 5). Also, the mucoadhesion study showed a stronger fluorescence signal (only images without quantitative analysis are provided in their publication) from thiol-functionalised silica nanoparticles compared to amino- and hydroxyl-functionalised counterparts (Figure 6). Additionally, the thiol-functionalised silica nanoparticles provided a sustained doxorubicin release, which was slower at the pH of artificial urine (6.1) compared to the pH of phosphate buffer solution (7.4) (~13% and 63% cumulative release after 48 h, respectively) [101]. The greater release from the thiol-functionalised silica nanoparticles may be due to the protonation of the amino groups of the β-cyclodextrin leading to the formation of positively charged rings around the mesopores of the nanoparticles. As doxorubicin is also positively charged, electrostatic repulsion will be present, which increases the size of the mesopores of the nanoparticles and facilitates the drug release [101].

**Figure 7:**
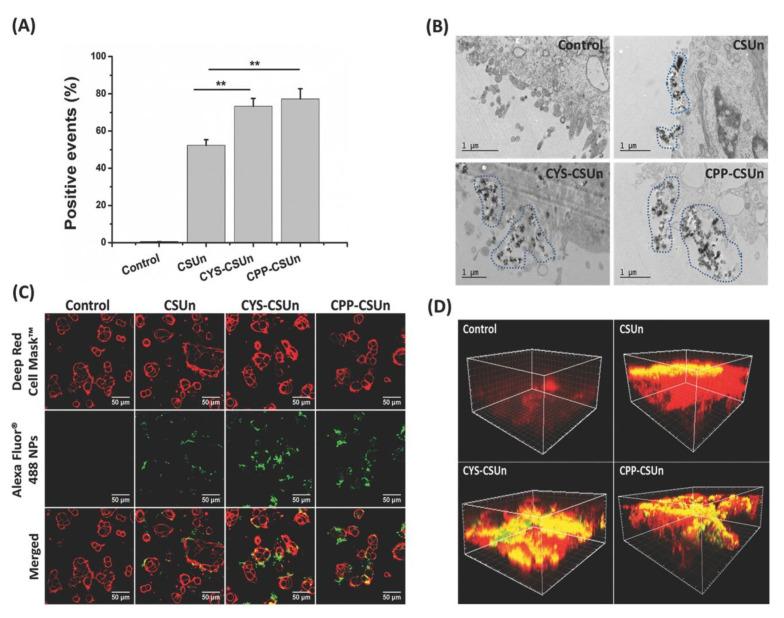
(**A**) Flow cytometry of cocultured Caco-2/HT29-MTX cells interacting with different silicon nanoparticles. **: the statistical significant difference (*p* < 0.01) between the CSUn and CYS-CSUn or CPP-CSUn nanoparticles. (**B**) TEM images of flat embedded ultrathin sections of cell monolayers interacting with different silicon nanoparticles. (**C**) Confocal microscopy images of different AlexaFluor ^TM^ (Life Technologies, USA) conjugated silicon nanoparticles interacting with Caco-2/HT29 coculture cells after a 3 h incubation at 37 °C; Red colour indicates cell membranes stained with CellMask^TM^ DeepRed (Life Technologies, USA); green colour indicates AlexaFluor ^TM^ conjugated nanoparticles; yellow colour indicates co-localization of nanoparticles and the cell membranes. (**D**) 3D confocal microscopy images of the cell monolayers interacting with different nanoparticles (red colour: mucus layer stained with wheat germ agglutinin (WGA)-AlexaFluor ^TM^ 594; green colour: AlexaFluor ^TM^ 488 labelled silicon nanoparticles; and yellow colour: co-localization of the mucus and the nanoparticles). Reprinted from Shrestha et al. [104] with permission of John Wiley & Sons.

Several studies have demonstrated that silicon nanoparticles that were uncoated, undecylenic acid-modified, thermally hydrocarbonised and porous interacted weakly with Caco-2/HT29-MTX (mono- and co-culture) cells, possibly due to the negatively charged surfaces of the nanoparticles. However, this interaction was enhanced when the silicon nanoparticles were coated with chitosan, through either physical adsorption or chemical conjugation, due to the adhesion of chitosan to the mucus secreted by HT29-MTX cells [102,103]. Shrestha et al. [104] modified such silicon nanoparticles with chitosan (to form CSUn nanoparticles). The nanoparticles were further modified with either cysteine or a cell penetrating peptide (CPP) to generate cysteine-functionalised (CYS-CSUn) or CPP-functionalised (CPP-CSUn) nanoparticles, respectively. They showed that both CYS-CSUn and CPP-CSUn nanoparticles enhanced the intestinal permeation of insulin through a triple co-culture of Caco-2, HT29-MTX and Raji B cells in a monolayer. In the case of CYS-CSUn nanoparticles, this was due to the presence of thiol groups in the structure of the nanoparticles, which form disulfide bonds with cysteine-rich domains of mucus glycoproteins. However, in the case of CPP-CSUn nanoparticles, the cell-penetrating ability of CPP was the major reason for the enhanced insulin permeation through the cells. This was confirmed by studying the interactions between the nanoparticles and the intestinal cells using flow cytometry, TEM, and confocal microscopy. It was found that both CYS-CSUn and CPP-CSUn nanoparticles showed stronger interactions with the surface of the intestinal cells compared to unmodified nanoparticles (Figure 7). Indeed, CPP-CSUn nanoparticles were internalised by the intestinal cells (Figure 7B,C). On the other hand, only CYS-CSUn nanoparticles enhanced the oral bioavailability of insulin in a type 1 diabetic rat model. The authors linked this to the possible degradation of the peptide layer of CPP-CSUn nanoparticles by luminal enzymes in the rat GIT, or the different nature of the mucus barrier of the in vivo model compared to the in vitro cell model [104]. The surface functionalisation is an interesting approach commonly used to facilitate the cellular internalisation of the silica nanoparticles and enhance their delivery efficiency [105,106,107,108].

**Figure 8:**
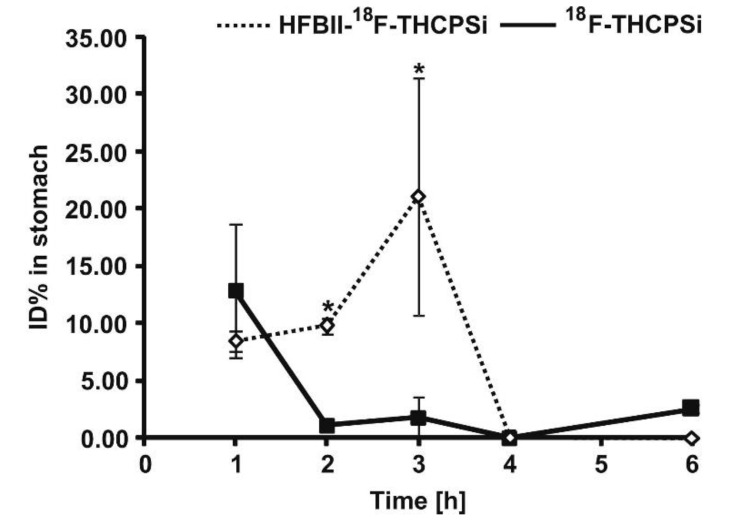
Comparison of gastric emptying time of HFBII-^18^F-THCPSi and non-functionalised^18^F-THCPSi nanoparticles in rats. ID% is the percentage of injected dose, which was calculated from the radioactivity of the gastric tissues. Data represent mean ± standard deviation (*n* = 3 per time point), *: *p* < 0.05. Reprinted from Sarparanta et al. [109] with permission of Elsevier.

Sarparanta et al. [109] reported that porous silicon nanoparticles which had been hydrophobin-functionalised, ^18^F-radiolabelled and thermally hydrocarbonised (HFBII-^18^F-THCPSi) showed stronger mucoadhesion in an in vitro model of human adenocarcinoma cells compared to non-functionalised ^18^F-THCPSi. This could be due to the electrostatic and hydrophobic interactions between specific amino acid residues of hydrophobin and the mucus components of the cells. Additionally, the authors suggested the formation of disulfide bonds between the cysteine residue of hydrophobin and the thiol groups of mucus glycoprotein. The in vivo study in rats using macroautoradiography showed that HFBII-^18^F-THCPSi nanoparticles were retained in the glandular part of the stomach for up to 3 h, due to their adhesion to the loosely bound mucus layer, followed by their transit into the small intestine. From the radioactivity measurements (Figure 8), it was concluded that the amount of HFBII-^18^F-THCPSi nanoparticles in the rat’s stomach was greater than the amount of the non-functionalised ^18^F-THCPSi nanoparticles. This indicated that HFBII-^18^F-THCPSi nanoparticles had a longer gastric emptying time than the non-functionalised ^18^F-THCPSi nanoparticles (Figure 8).

The organ-specific affinity of functionalised silica nanoparticles has also been demonstrated by other researchers. For example, in an in vivo study in mice, Desai et al. [110] showed that polyethylene imine (PEI)-functionalised silica nanoparticles had a greater affinity for the small intestine, whereas combined PEG-PEI-functionalised silica nanoparticles had a greater affinity for the colon. Such types of nanoparticles have potential applications in the design of targeted drug delivery systems for drugs like antibiotics and anticancer agents that target the GIT.

The effect of hydrophilic polymers on the interaction of silica nanoparticles with mucin was also investigated by other researchers. Andreani et al. [111] revealed that both alginate- and chitosan-coated silica nanoparticles interacted strongly with mucin, as evident from the reduction in their zeta potential upon dispersion in a mucin solution. These results showed that alginate- and chitosan-coated silica nanoparticles are mucoadhesive. In contrast, both non-coated and PEG-coated nanoparticles showed a weak interaction. This may indicate the ability of non-coated and PEG-coated silica nanoparticles to diffuse into the mucus network, i.e., they are non-mucoadhesive and therefore not trapped in the mucus gel.

Liu et al. [112] studied mesoporous silica nanoparticles as a dual-drug loaded carrier for a hydrophobic (indomethacin) and a hydrophilic (human peptide, PYY3-36) compound. They found that the presence of PYY3-36 in the indomethacin/PYY3-36-loaded silica nanoparticles increased the permeation of both indomethacin and PYY3-36 through co-cultured Caco-2/HT29 cell monolayers. They related this to the presence of mucus secreted by the HT29 cells, leading to interactions between cell-silica nanoparticles that resulted in a high local drug concentration close to the cellular monolayers.

Several other studies have demonstrated the potential of silica nanoparticles in transmucosal drug delivery. Table 1 illustrates the use of silica nanoparticles for the delivery of various drugs with their routes of administration, the method of evaluation of mucoadhesion/mucus penetration, surface chemistries, and advantages.

## 7. Safety Considerations and Biodistribution of Silica Nanoparticles

The safety and biodistribution of silica nanoparticles are controversial and have been found to be highly dependent on the size, shape, surface properties, cell type, animal species, dose and the method of administration. Using the everted gut sac method, Yoshida et al. [119] demonstrated that silica nanoparticles of various sizes (70, 300, and 1000 nm) and surface functionalities (carboxyl or amino groups) were absorbed by the rat small intestine. However, they observed no abnormalities in the mice after a 28-day oral exposure to these nanoparticles, as indicated by histopathology examination of the liver, kidney, brain, lung, spleen, heart, stomach and intestine and haematological analysis. Using TEM, Yoshida et al. [120] found that following nasal administration of silica nanoparticles in mice (20 μL, at 500 μg/mouse daily for 7 days), the particles of 30, 70, and 100 nm were absorbed by the nasal mucosa and detected in the nasal cavity, lung and liver. On the other hand, 1000 nm particles were detected in the nasal cavity and lung, whereas 300 nm particles were only detected in the lung. Neither the 300 nm nor the 1000 nm particles were detected in the liver. Yoshida et al. [120] did not provide any explanation for the difference observed in the biodistribution of these nanoparticles, but suggested that TEM was only a qualitative method and thus no quantitative data could be obtained. They also hypothesised that the larger nanoparticles (300 nm and 1000 nm) degraded in the biological environment into smaller nanoparticles [120], which could explain why these nanoparticles were not detected in the liver. However, they did not show any experimental data to support the fact that these nanoparticles degrade in the biological environments as the biodegradability of silica nanoparticles is controversial and it mainly depends on the type of the nanoparticles [121,122,123]. In terms of toxicity, only the 30 nm and 70 nm nanoparticles prolonged the bleeding time of mice compared to the control. No adverse biological effects were observed with the other nanoparticles [120].

In rats, subcutaneous injection of mesoporous silica particles (150–4000 nm) produced no apparent toxicity. However, intravenous and intraperitoneal injections in mice led to the death of the animals, possibly due to pulmonary thrombosis [124]. Oral and ocular administration of nonporous silica nanoparticles to rats for 12 weeks was found to be safe [125].

Li et al. [126] observed possible renal impairment with sphere-like mesoporous silica nanoparticles but not with rod-like mesoporous silica nanoparticles when orally administered to mice. They also reported that the silica nanorods mainly accumulated in the liver and spleen of the mice, whereas the silica nanospheres were mainly found in the spleen. Some other investigators have revealed the impact of silica nanoparticle shape on their toxicity, biodistribution, and biocompatibility [8,127,128]. Bukara et al. [129] found that ordered mesoporous silica nanoparticles are well tolerated by human volunteers and the nanoparticles improved the oral bioavailability of fenofibrate.

It can be concluded that the toxicity of the silica nanoparticles mainly depends on the chemical composition, the size, the shape and the routes of administration of the nanoparticles [3,128]. Different mucosal surfaces show different barrier properties to various silica nanoparticles. In other words, silica nanoparticles do not penetrate mucosal tissues with different pore sizes of the mucus gel, mucus thickness, and pH to the same extent.

## 8. Potential for Future Research

Since the pioneering studies by Nagai et al. [71,72], hydrophilic polymers have been traditionally used in the design of mucoadhesive dosage forms for transmucosal drug delivery. Their mucoadhesive properties were related to the ability of functional groups to interact with mucins via electrostatic interactions and hydrogen bonding as well as to the ability of polymeric macromolecules to diffuse into the mucus gel and form interpenetrating layers [130,131]. In recent years, a significant progress has been achieved in functionalisation of various polymers to make them more mucoadhesive. Several synthetic strategies have emerged including functionalisation of polymers with thiol-, catechol-, boronate-, acrylate-, methacrylate-, maleimide-, and *N-*hydroxy(sulfo)succinimide ester-groups [81].

Silica nanoparticles simply composed of silicon dioxide do not exhibit substantial mucoadhesive properties. However, due to the numerous possibilities for their surface functionalisation it is possible to make them mucoadhesive. Some studies demonstrating the mucoadhesive properties of silica nanoparticles through the chemical functionalisation of their surfaces already emerged, which include thiolation and decoration with amino-groups. More research is expected in this area considering the substantial expansion in the chemistries favouring mucoadhesion that have emerged in recent years in the studies of mucoadhesive polymers [81]. Advances in this area are also expected not only with silica, but also with other inorganic and hybrid colloids, such as metals (e.g., gold and silver) and other inorganic oxide nanoparticles (e.g., titanium dioxide). Some progress in surface-functionalised gold nanoparticles has recently been reported [132].

Another area for the potential development and application of silica nanoparticles in transmucosal drug delivery arises from a recently emergent interest in mucus-penetrating particles. Hanes et al. [133,134,135] demonstrated the excellent potential of PEGylated nanoparticles of polymeric nature for transmucosal drug delivery. Some other studies also reported the potential of PEGylated materials in the formulation of mucus-penetrating nanoparticles [136,137,138,139]. Some non-ionic polymers other than polyethyleneglycol were also reported to exhibit mucus-penetrating properties [82]. Our research group has recently demonstrated the possibility of making silica nanoparticles more mucus-and tissue-penetrating via their PEGylation and POZylation [96,97,99,100]. Due to the relative ease of silica surface functionalisation with polymers, some further research is expected in the development of novel mucus-penetrating silica-based particles.

Further studies on the safety in human, reproducibility, stability and scalability of the silica nanoparticles are expected. Numerous studies reported the toxicity profiles of silica nanoparticles in vitro cells or animal models [140,141,142,143,144,145]. However, to date, only one study confirmed that silica nanoparticles are safe in healthy humans [129]. Silica nanoparticles are usually prepared at the small scale in research laboratories with the proper control of various important formulation parameters, including the pH, temperature, oxygen level, etc., which usually results in a reproducible control of the nanoparticles size, polydispersity index, and shapes. However, the preparation of silica nanoparticles on industrial scale is likely to be a challenging task due to the difficulties in controlling the formulation parameters. Advances in characterisation techniques can potentially allow better control on the size and the long-term stability of the silica nanoparticles which can improve the reproducibility of the nanoparticles. Studies on the aforementioned areas may lead to the translation of these promising drug delivery systems from the bench to the clinic.

## 9. Conclusions

Silica nanoparticles are promising drug nanocarriers for transmucosal drug delivery, due to their relatively simple methods of preparation, control over particle size and shape, high drug loading, and controlled drug delivery. Different silica nanoparticles could be synthesised bearing various functional groups. Some surface functional groups (for example, amino or thiol groups) could make the nanoparticles more mucoadhesive. These groups could also be used to functionalise the nanoparticle with polymers. PEGylation and POZylation of silica nanoparticles could make them mucus-penetrating. Although silica nanoparticles have generally been found to be relatively safe, a few studies have raised some safety concerns. These suggest further pre-clinical investigations to explore their potential applications in transmucosal drug delivery.

## Figures and Tables

**Table 1:** Some examples of transmucosal drug delivery using silica nanoparticles in the literature.

Drugs	Uses	Routes of Administration	Models for Mucoadhesion and Therapeutic Evaluation	SURFACE Chemistries	Advantages	References
5-amino salicylic acid	Inflammatory bowel disease	Oral	In vivo using mice	Coated with chitosan	Delayed drug release and targeted delivery to the inflamed tissues	[113]
Glucagon like peptide-1	Type 2 diabetes mellitus	Oral	In vitro using intestinal cells	Coated with chitosan	Chitosan coated silica nanoparticles provided high drug loading capacity, sustained drug release and enhanced drug permeation	[102]
Curcumin	Neurodegenerative diseases	Nasal	In vitro using olfactory neuroblastoma cells	No coating	Targeting the brain, better chemical stability of the loaded drug	[114]
Doxorubicin	Bladder Cancer	Intravesical	In vitro porcine bladder mucosa	Poly(amidoamine) dendrimers	Controlling the level of surface layer though a layer-by-layer grafting method, Enhanced retention in bladder mucosa, Sustained drug release which was triggered in acidic environment	[115]
Paclitaxel (as a model drug)	Cancer	Oral	Incubating particles in mucin suspension, Caco-2 cells, In vivo studies in rats	Quantum dots doped hollow silica nanoparticles were first coated with cationic cell-penetrating peptides and then with a mucus-inert hydrophilic succinylated casein layer	Protects the drug from gastric acid, Degrades and then releases the drug in small intestine, Enhanced mucus-penetration, Strong interaction with epithelial membranes and a 5-fold increase in cellular uptake, Enhanced absolute bioavailability and in vivo antitumor activities	[116]
Lopinavir (as a model drug)	AIDS	Oral	Caco-2/HT29 cells, Everted gut sac method, In vivo bio-distribution studies and pharmacokinetic studies in rats	The core silica nanoparticles were coated with a middle layer of a cell-penetrating peptide and an outer layer of a thiolated polymer	Enhanced mucoadhesion and absorption through epithelial cells simultaneously, Enhanced oral bioavailability	[117]
Ovalbumin (as a model antigen)	Vaccination	Oral	Mucin binding assay	3-aminopropyltriethoxysilane, poly(methyl methacrylate) (PMMA), PEG, chitosan	PMMA, PEG and chitosan modified nanoparticles provided sustained drug release, PEG and chitosan modified nanoparticles showed high encapsulation efficiency, Remained intact in simulated gastric and intestinal fluids, Showed enhanced mucoadhesion	[118]
